# External Validation and Calibration of IVFpredict: A National Prospective Cohort Study of 130,960 In Vitro Fertilisation Cycles

**DOI:** 10.1371/journal.pone.0121357

**Published:** 2015-04-08

**Authors:** Andrew D. A. C. Smith, Kate Tilling, Debbie A. Lawlor, Scott M. Nelson

**Affiliations:** 1 Medical Research Council Integrative Epidemiology Unit, the University of Bristol, Bristol, United Kingdom; 2 School of Social and Community Medicine, University of Bristol, Bristol, United Kingdom; 3 School of Medicine, University of Glasgow, Glasgow, United Kingdom; Institute of Zoology, Chinese Academy of Sciences, CHINA

## Abstract

**Background:**

Accurately predicting the probability of a live birth after in vitro fertilisation (IVF) is important for patients, healthcare providers and policy makers. Two prediction models (Templeton and IVFpredict) have been previously developed from UK data and are widely used internationally. The more recent of these, IVFpredict, was shown to have greater predictive power in the development dataset. The aim of this study was external validation of the two models and comparison of their predictive ability.

**Methods and Findings:**

130,960 IVF cycles undertaken in the UK in 2008–2010 were used to validate and compare the Templeton and IVFpredict models. Discriminatory power was calculated using the area under the receiver-operator curve and calibration assessed using a calibration plot and Hosmer-Lemeshow statistic. The scaled modified Brier score, with measures of reliability and resolution, were calculated to assess overall accuracy. Both models were compared after updating for current live birth rates to ensure that the average observed and predicted live birth rates were equal. The discriminative power of both methods was comparable: the area under the receiver-operator curve was 0.628 (95% confidence interval (CI): 0.625–0.631) for IVFpredict and 0.616 (95% CI: 0.613–0.620) for the Templeton model. IVFpredict had markedly better calibration and higher diagnostic accuracy, with calibration plot intercept of 0.040 (95% CI: 0.017–0.063) and slope of 0.932 (95% CI: 0.839–1.025) compared with 0.080 (95% CI: 0.044–0.117) and 1.419 (95% CI: 1.149–1.690) for the Templeton model. Both models underestimated the live birth rate, but this was particularly marked in the Templeton model. Updating the models to reflect improvements in live birth rates since the models were developed enhanced their performance, but IVFpredict remained superior.

**Conclusion:**

External validation in a large population cohort confirms IVFpredict has superior discrimination and calibration for informing patients, clinicians and healthcare policy makers of the probability of live birth following IVF.

## Introduction

For a patient or couple considering in-vitro fertilisation (IVF) the most important prognosis is that of a live birth, and for the clinician advising them it is important to be able to provide an accurate assessment of that prognosis [1]. For policy makers, precise estimates of prognosis are essential to model the population burden of infertility and treatment, and to inform cost-effective healthcare provision [2,3]. As clinicians’ assessment of prognosis are widely varied [4,5], several prediction models have been developed to give the prognosis of live birth based on patient and couple characteristics and measurements, in order to better inform patients and clinicians [6–14]. Many of these include measurements that would not be available prior to commencing the first cycle of IVF, and would hence have limited ability to inform decisions, and/or have not been externally validated.

Two prediction models have been developed using data from the UK [7,12], where there is a statutory legal requirement to maintain a national record of every initiated IVF cycle and its outcome. The first of these, the Templeton model [7], has been widely used [1], externally validated [12,15–18], and recommended as the best model in systematic reviews [1,15]. However, it was developed using data from couples who received IVF two decades ago, when successful live birth rates were considerably lower than currently, and before the introduction of intra-cytoplasmic sperm injection (ICSI), which has transformed the treatment of male infertility [19]. Recently, we developed IVFpredict in the largest IVF prediction study to date (144,018 cycles) and added prognostic characteristics, including ICSI, to those used in the Templeton model [12]. We demonstrated that IVFpredict had superior discrimination and calibration to the Templeton model [12], and this model is increasingly used internationally. A recent Dutch study of 5,176 treatment cycles has externally validated IVFpredict but also showed that the Templeton model had similar discrimination and calibration [18]. However, in addition to having a relatively small sample size, that study only included couples with primary infertility and excluded those who used donor eggs. The authors acknowledged that these limitations were particularly likely to adversely affect the calibration of the IVFpredict model. Furthermore, the authors were only able to examine prediction of pregnancy, rather than live birth, though a correction factor was used in an attempt to produce an estimated live birth rate.

The live birth rate from IVF has continued to increase since the development of IVFpredict [20], and the mix of patients referred for IVF has changed [17]. Hence it is possible that it, as well as the Templeton model, will need to be updated for accurate use in a new cohort [15]. Indeed in the Dutch study, described above, both of the Templeton and IVFpredict models performed better if adjustments were made for pregnancy/live birth success rate in each cohort [18]. This raises the issue of whether, even with good prediction models that have been externally validated, their application in practice has to take account of success rates in the particular population that the couple, their clinician and policy makers might consider they belong to. Lastly, IVFpredict used broad female age categories, as during its development we were only provided with data that allocated each treatment cycle to the woman’s age category publicly reported by the Human Fertilisation and Embryology Authority (HFEA). This has been criticised as likely to result in marked over-fitting of our model [18]. As female age is the strongest predictor of live birth success [7], it is possible that female age alone could accurately predict successful outcome, which would be a simple useful tool for all patients, clinicians and policy makers.

The purpose of this study is to perform validation of the IVFpredict and Templeton prediction models on a new UK cohort of IVF cycles. We aim to compare the predictive ability of the two models and to examine how much each prediction model requires updating in a new sample where success rates vary from those in the cohort used to originally develop the model. We also aim to quantify the value, in terms of predictive ability, of including covariates other than female age in the prediction models. In the validation sample used here we have female age in years at each cycle, and we also explore whether the predictive ability of IVFpredict is improved by using female age as a continuous variable. Since the quantity and quality of validation studies has been criticised [21], we aim to make use of a large validation sample size and perform all of the validation measures recommended in recent literature [21–23].

## Materials and Methods

### Ethics statement

The HFEA provided ethical approval for this study. All data were analyzed anonymously.

### Data

The HFEA has a statutory duty to collect and record information about every assisted conception treatment in the UK. By law, every treatment centre must report certain couple characteristics, treatment details and outcomes for every initiated IVF cycle. The HFEA provided a database of all IVF cycles in the UK in the period 2003–2010. A cycle of IVF was defined as an initiated ovarian stimulation or planned fresh or frozen embryo transfer. Since IVFpredict was built using data from 2003–2007, we restricted the validation sample used here to cycles occurring in 2008–2010. Data on live births for cycles initiated in 2011 onwards were not completely available. We used the same exclusion criteria as our previous study where we developed IVFpredict and compared it with the Templeton model, excluding treatments that are not IVF (i.e. involve donor insemination or gamete/zygote intra-fallopian transfer), involve the storage or donation of eggs, or use frozen embryo transfer [12]. IVFpredict cannot give a prediction for women aged more than 50 years, so cycles from these women were excluded along with cycles for which data on the duration of infertility were missing.

### Prediction models

The Templeton and IVFpredict models use a linear predictor that differs with patient and cycle characteristics. This is converted into the predicted probability of a live birth using the logistic transformation. Equivalently, the linear predictor equals the log odds of a live birth.

### IVFpredict

The variables used by IVFpredict are: female age (categorized as 18–34, 35–37, 38–39, 40–42, 43–44 and 45–50 years), duration of infertility (less than 1, 1–3, 4–6, 7–9, 10–12 and more than 12 years attempting to conceive), cause of infertility (tubal, ovulatory, endometriosis, cervical, male or combined), number of previous IVF cycles and number of previous unsuccessful IVF cycles, pregnancy history, type of ovulation induction, whether ICSI was used, and whether donor or the patient’s own eggs were used [12]. Gonadatropins are now recognized as the optimal agent for induction of multifollicular growth for IVF [24]. In our validation sample the agent used was not recorded for 128,438 cycles (98.1%), but it was recorded as gonadatropins in 2,511 cycles (99.6%) where it was reported. We therefore assumed that all cycles used gonadatropins and gave the IVFpredict linear effect attributed to this to all cycles. The interaction terms included in IVFpredict: between female age and duration, female age and egg source, ICSI and cause of infertility, and ICSI and number of previous IVF cycles, were included here.

### Templeton model

The Templeton model uses female age (considered as a continuous variable with its effect on the log odds represented by a cubic curve), duration of infertility (less than 4, 4–6, 7–12 and more than 12 years), number of previously unsuccessful IVF cycles, pregnancy history, and tubal cause of infertility [7]. The original Templeton model does not include any interactions.

### Female age-alone models

We wished to compare both the Templeton and IVFpredict models with a model that predicts live birth outcome using female age alone. However, there was no clear candidate for such a model in the literature, and it would not be appropriate to develop one using the validation data as its performance would be biased in the validation data. Instead, we considered a model in which the predicted probability of live birth decreased as female age increased. The exact shape of the relationship between female age and predicted probability of live birth does not affect the discriminatory power of this model, provided we assume this monotonic relationship. We also considered a model that used the HFEA age categories to inform prediction, again with decreasing live birth rate with increasing female age, in order to measure the reduction in discriminatory power associated with using female age as a categorical rather than continuous variable.

### Statistical methods

We used several methods, recommended in recent reviews [21–23], to assess the validity of both models and compare their performance in the validation sample. These methods may be thought of as assessing discriminatory power, calibration, or a combination of these two properties of a prediction model. Discriminatory power refers to the ability of a prediction model to discriminate between successful and unsuccessful outcomes. This was assessed using the area under the receiver-operator curve (AUROC). In this context the AUROC is the probability that a model will predict a better prognosis for a randomly-selected cycle that resulted in a live birth than a randomly-selected cycle that did not result in a live birth. We compared the AUROC of IVFpredict with the reported AUROC and confidence intervals (CIs) in the IVFpredict development sample [12], using a Wald test, assuming independence between the development and validation samples.

Calibration refers to the similarity between the observed and predicted live birth rate in groups of cycles. We assessed general calibration using calibration plots, which average the observed and predicted live birth rate over deciles of the linear predictor. In a calibration plot, the observed live birth rate is plotted against predicted live birth rate, and perfect calibration is indicated by a straight line, with a gradient of one, through the origin. We used linear regression to estimate the intercept and slope of the closest-fitting straight line to the points on the calibration plot, and assessed departures from perfect calibration using the Hosmer-Lemeshow test [25 p147–156]. We also assessed calibration over patient and cycle characteristics by comparing the observed and predicted live birth rates by female age, egg source, duration of infertility, number of previously unsuccessful IVF cycles, previous live birth from IVF, cause of infertility, and use of ICSI. Here, departures from perfect calibration were assessed with Pearson’s chi-squared test and p-values were considered against a Bonferroni-corrected threshold that ensured the family-wise error rate for all tests of calibration over patient and cycle characteristics was not greater than 5%.

The Brier score is a combination of the discriminatory power and calibration of a prediction model [26 p284–287]. We calculated modified Brier scores, being the Euclidean distance between observed and predicted live birth rates over deciles of the linear predictor, and scaled them by dividing by the sample variance of the observed live birth rate. The scaled modified Brier scores can be decomposed into measures of reliability and resolution. The reliability is a goodness-of-fit statistic and is related to the Hosmer-Lemeshow test for calibration. The resolution measures the range of probabilities that the prediction model can handle, with a higher value indicating that the model can predict over a larger range of probabilities, which given IVF treatment is now used in couples ranging from complete infertility to marginally reduced fertility (or even normal fertility), is desirable [20]. When the overall observed and predicted live birth rates are equal, as in the updated models, the scaled Brier score is the proportion of variation in observed live birth rates not explained by the prediction model.

It has been suggested that prediction models can be compared by evaluating how many patients are correctly reclassified from one treatment recommendation to another [23]. Since both the IVFpredict and Templeton models (and other models for predicting successful pregnancy/live birth outcome) do not give thresholds of prognosis or treatment recommendations, only the predicted probability of live birth, it is not possible to compare them in this way. However it is possible to assess, for each cycle, which prediction model gave a prognosis closer to the truth. We did this by cross-tabulating the number of cycles resulting in live births against the prediction model that gave a higher probability of success to that cycle. We also calculated a continuous version of the net reclassification index [27], which was calculated as twice the proportion of live births given a higher prognosis by IVFpredict minus twice the proportion of cycles not resulting in a live birth given a higher prognosis by IVFpredict.

For each of the IVFpredict and Templeton models we assessed two different predictions. The first was calculated using the original values for the linear predictors. The second prediction was updated for the validation sample by adding a constant term to the linear predictor, calculated numerically by trial-and-improvement to ensure that the average observed and predicted live birth rates in the whole sample were equal. The simpler method of adjusting the linear predictor by adding the difference between the observed and predicted log odds of live birth [28], would not achieve this, nor would adding the difference between the observed log odds of live birth in the validation sample compared with the development sample, as was done in the Dutch study [18]. A more complicated method is to refit the prediction model to the validation sample by re-running the logistic regression [15,17], or to use a calibration plot to find an adjusted prediction (vertical value) corresponding to each predicted prognosis (horizontal value). A drawback of these methods is that they cause the validation sample to become a new development sample, hence the refit model requires further validation, and therefore we did not use these methods. Updating the models, using any of the methods above, would affect their calibration but not their discriminatory power.

All statistical analyses were performed using Stata version 12 (StataCorp LP).

## Results

**Fig 1:**
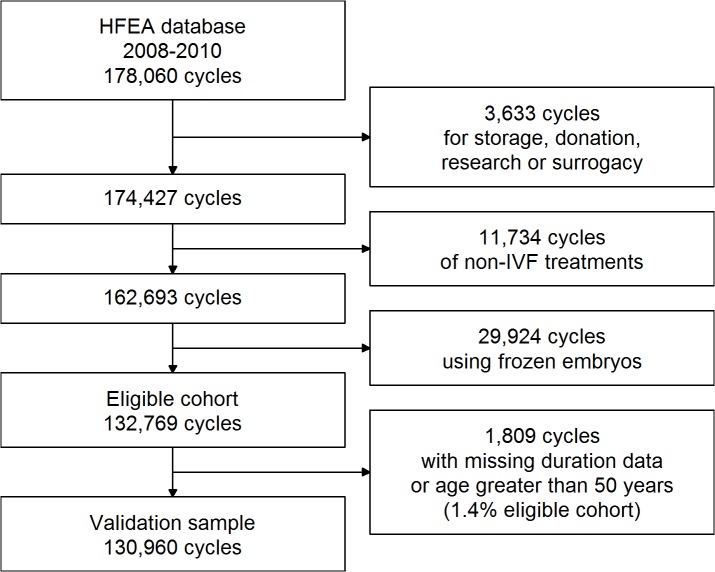
Inclusion/exclusion criteria and formation of the validation sample.

There were 132,796 eligible IVF cycles that took place in the UK between 2008 and 2010. It was not possible to calculate a predicted probability of live birth for 1.4% of these cycles, due to missing information on duration of infertility or patient age greater than 50 years. There were a remaining 130,960 cycles available for validation; details of the formation of the validation sample are given in Fig. 1. Table 1 shows the characteristics of the treatment cycles in this study. Over half (51.7%) of cycles involved ICSI, and the proportion of cycles involving donor eggs increased with female age. There were 33,553 live births, giving a successful live birth rate of 25.6% per cycle in this cohort.

**Table 1:** Characteristics of patients and treatment in the validation sample, consisting of 130,960 IVF cycles.

Characteristic	Number in sample	Percentage of sample	Percentage using donor eggs
Live births	33,553	25.6%	
Patient age at treatment (years)			
18–34	53,556	40.9%	1.0%
35–37	31,240	23.4%	1.6%
38–39	21,348	16.3%	2.2%
40–42	18,143	13.9%	4.8%
43–44	4,688	3.6%	14.0%
45–50	1,985	1.5%	49.0%
Duration of infertility (years)			
0	3,125	2.4%	
1–3	59,996	45.8%	
4–6	44,400	33.9%	
7–9	14,485	11.1%	
10–12	5,560	4.3%	
13-	3,394	2.6%	
Previous live birth			
Yes	23,444	17.9%	
No	107,516	82.1%	
Previous unsuccessful IVF cycles			
0	83,838	64.0%	
1	25,051	19.1%	
2	11,862	9.1%	
3-	10,209	7.8%	
Treatment type			
IVF	63,245	48.3%	
IVF plus ICSI	67,715	51.7%	
Source of egg			
Patient	126,962	97.0%	
Donor	3,998	3.0%	
Cause of infertility			
Unexplained	45,928	35.1%	
Tubal only	15,244	11.6%	
Ovulatory only	8,526	6.5%	
Endometriosis only	5,164	3.9%	
Cervical only	5	0.0%	
Male cause only	43,663	33.3%	
Multiple causes	12,430	9.5%	

### Discrimination

Table 2 shows the AUROC for the IVFpredict and Templeton models. Despite strong statistical evidence for a difference in discrimination, with IVFpredict performing better than Templeton, the AUROC values were similar for the two models, with both showing good discrimination. IVFpredict had slightly poorer discrimination in this validation cohort than in the original cohort, and both models had better discrimination than a model based on female age alone as a continuous variable. The categorisation of female age resulted in a decrease in AUROC of 0.004, which was 0.7% of the AUROC of IVFpredict, suggesting that there would be a very small increase in AUROC if IVFpredict were to be redesigned to use female age as a continuous variable.

**Table 2:** Area under the receiver-operator curve for IVFpredict and Templeton models, and female age alone, for predicting live birth from 130,960 IVF cycles.

	AUROC (95% CI)	Difference from row above
IVFpredict, development sample*	0.635 (0.630, 0.637)	
IVFpredict	0.628 (0.625, 0.631)	0.006 (*p* = 0.019)
Templeton model	0.616 (0.613, 0.620)	0.012 (*p* < 0.001)
Continuous female age**	0.610 (0.606, 0.612)	0.007 (*p* < 0.001)
Categorical female age***	0.604 (0.601, 0.608)	0.004 (*p* < 0.001)

* Based on 144,018 cycles occurring in the UK between 2003 and 2007 [12].

** Model predicting decreasing probability of live birth with increasing female age.

*** Model predicting decreasing probability of live birth with increasing female age categories (18–34, 35–37, 38–39, 40–42, 43–44 and 45–50 years).

### Calibration

**Fig 2:**
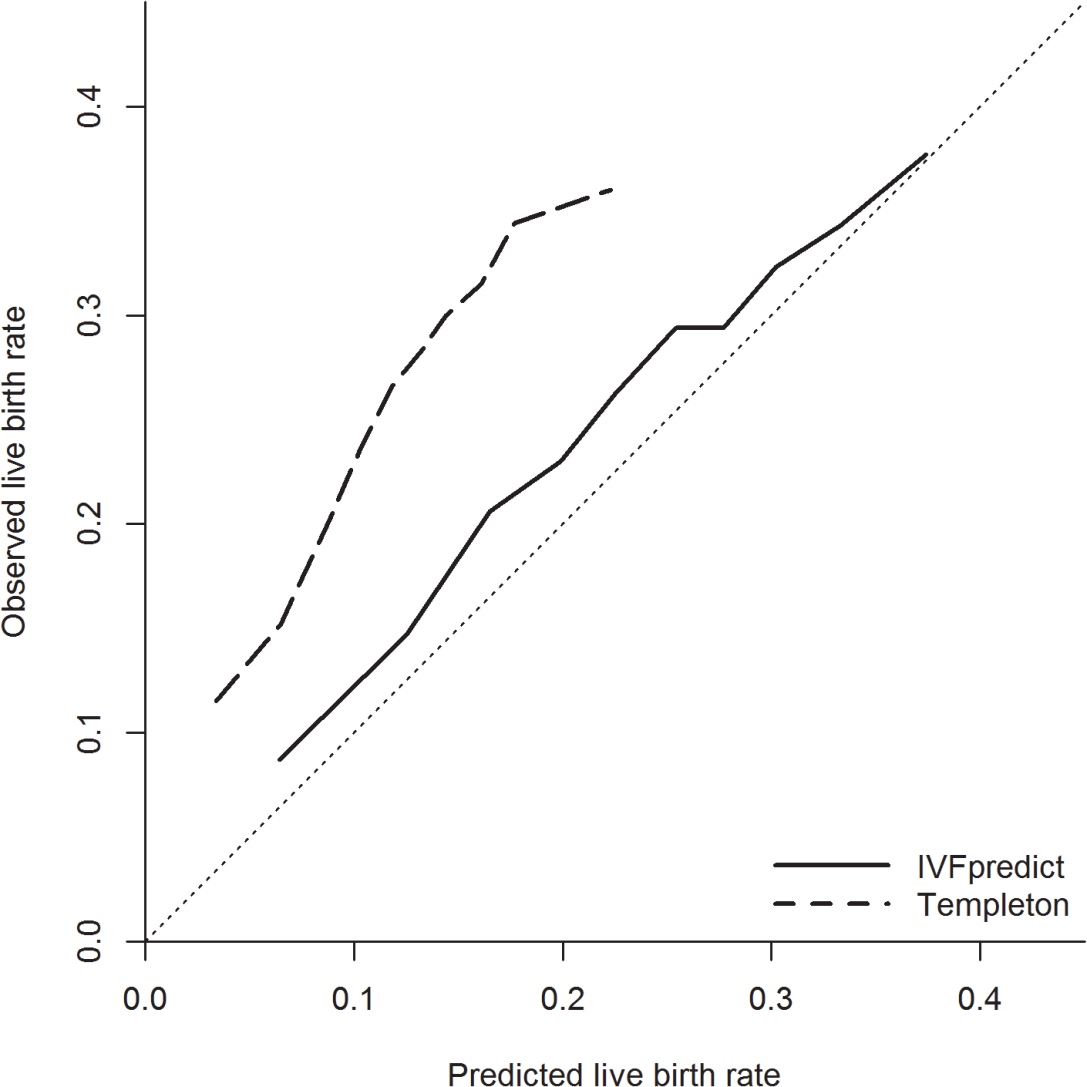
Calibration plot for the IVFpredict and Templeton models. Based on 130,960 IVF cycles. Hosmer-Lemeshow test statistics: p<0.001. Solid line, IVFpredict model; dashed line, Templeton model; dotted, diagonal line, perfect prediction (reference).

Fig. 2 shows calibration plots for the IVFpredict and Templeton models, showing observed versus expected live birth rates per decile of the linear predictor of each model. Perfect calibration is depicted by the reference line in Fig. 2, which has an intercept of 0 and a slope of 1. In contrast, the IVFpredict calibration plot had an intercept of 0.040 (95% CI: 0.017–0.063) and slope of 0.932 (95% CI: 0.839–1.025), and the Templeton model calibration plot had an intercept of 0.080 (95% CI: 0.044–0.117) and slope of 1.419 (95% CI: 1.149–1.690). Both models underestimate the live birth rate—this is seen in Fig. 2 as the calibration curves lie above the reference line—indicating that observed live birth rates were above those predicted. This is particularly marked in the Templeton Model. The actual differences between observed and predicted live birth rates are given in S1 Table.

### Effect on calibration of updating the prediction models

Both models required updating to ensure that the predicted live birth rate was, on average, equal to the observed live birth rate in the validation sample. IVFpredict was updated by adding 0.1396 to the linear predictor, thus increasing the predicted odds of live birth by a factor of 1.15. The Templeton model required a greater change: adding 0.9269 to the linear predictor, increasing the predicted odds of live birth by a factor of 2.53.

**Fig 3:**
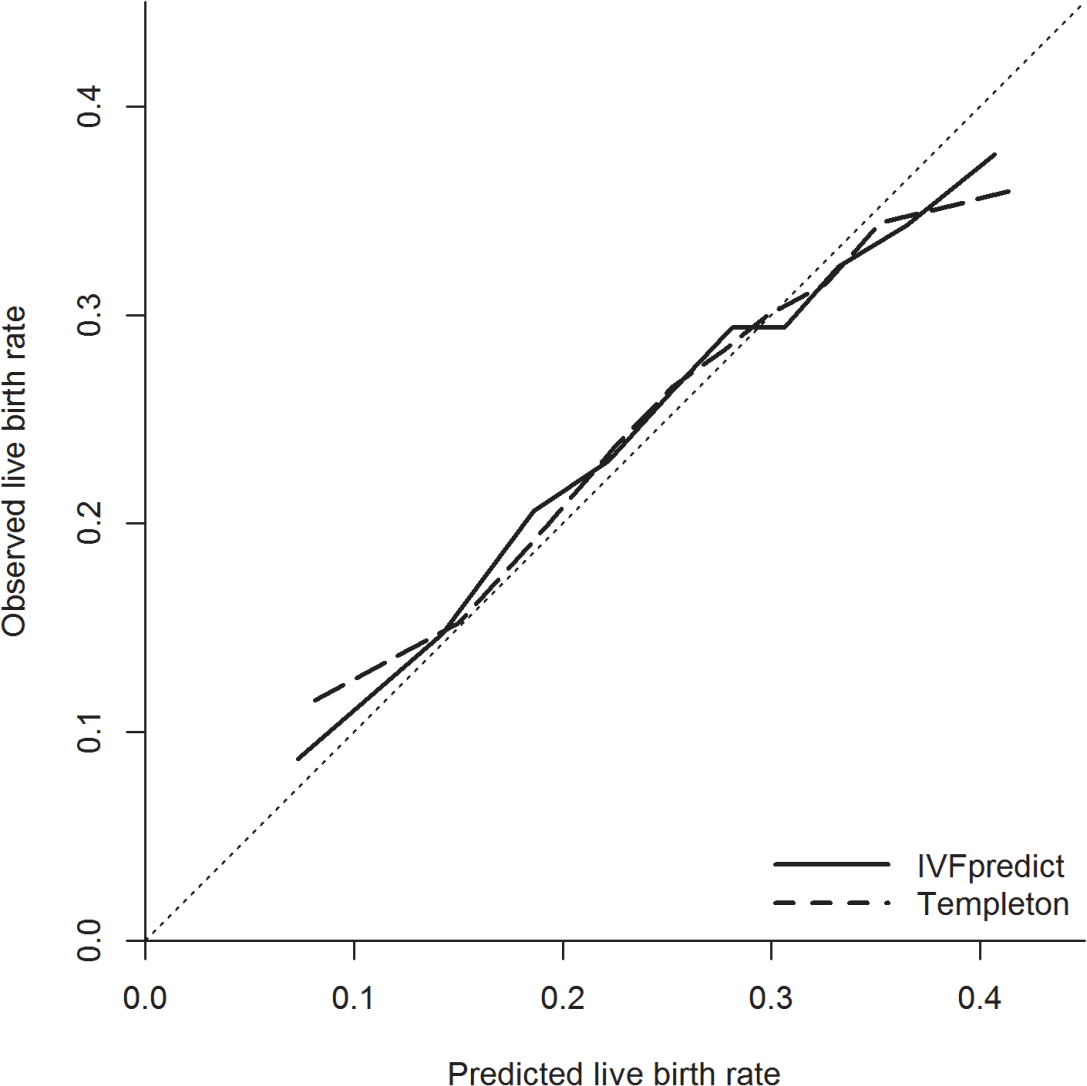
Calibration plot for updated IVFpredict and Templeton models. Based on 130,960 IVF cycles. Hosmer-Lemeshow test statistics: p<0.001. Solid line, updated IVFpredict model; dashed line, updated Templeton model; dotted, diagonal line, perfect prediction (reference).

Calibration plots for both updated models are shown in Fig. 3 (S2 Table). Both models showed a closer adherence to the reference line, although the Hosmer-Lemeshow test still showed strong statistical evidence of imperfect calibration in both models. As in the validation models without updating, calibration was better for the IVFpredict than the Templeton model. The IVFpredict calibration plot had a slope of 0.867 (95% CI: 0.789–0.946) whilst the Templeton calibration plot had a slope of 0.804 (95% CI: 0.697–0.912). Slopes less than 1 demonstrate that both updated models overestimated the live birth rate in couples with good prognosis, and underestimated the live birth rate in couples with poor prognosis. Since both models have been updated, the average observed and predicted live birth rates are equal, so the intercepts of the calibration plots give no additional information about the calibration of each updated model.

### Calibration by different couple characteristics

Table 3 shows the calibration of both updated models by couple characteristics. IVFpredict did not show differential calibration with respect to cause of infertility and treatment type, whilst the Templeton model showed differential calibration over all variables considered in Table 3. Both models underestimated the live birth rate in patients using donor eggs, particularly in women aged 38 years or older, but this was more marked in the updated Templeton model. In women aged 45 years or older using donor eggs the predicted live birth rate from the Templeton model was only 8% (95% CI: 7–9%) of the observed live birth rate, whereas from IVFpredict it was 84% (95% CI: 77–92%). The Templeton model overestimated the live birth rate in women aged 38 years or older using their own eggs and women with a tubal cause of infertility, whilst underestimating the live birth rate in women who had not previously had a live birth from IVF, and couples using ICSI.

**Table 3:** Observed and predicted live birth rates from updated IVFpredict and Templeton models stratified by characteristics of patients and treatment, in 130,960 IVF cycles.

		IVFpredict		Templeton	
	Observed live birth rate	Predicted live birth rate (SD)	Ratio predicted to observed (95% CI)	Predicted live birth rate (SD)	Ratio predicted to observed (95% CI)
Female age (years) patient eggs					
18–34	0.326	0.330 (0.060)	1.013 (1.000, 1.025)	0.327 (0.058)	1.004 (0.992, 1.016)
35–37	0.273	0.273 (0.056)	0.999 (0.980, 1.017)	0.272 (0.066)	0.996 (0.978, 1.014)
38–39	0.198	0.202 (0.047)	1.020 (0.992, 1.047)	0.210 (0.059)	1.062 (1.033, 1.091)
40–42	0.129	0.123 (0.033)	0.949 (0.913, 0.986)	0.148 (0.051)	1.140 (1.096, 1.184)
43–44	0.050	0.045 (0.015)	0.907 (0.785, 1.029)	0.079 (0.029)	1.574 (1.362, 1.785)
45–50	0.020	0.022 (0.014)	1.120 (0.631, 1.609)	0.036 (0.016)	1.827 (1.031, 2.623)
		*p* = 0.02		***p* < 0.001**	
Female age (years) donor eggs					
18–34	0.301	0.317 (0.067)	1.054 (0.917, 1.191)	0.322 (0.064)	1.070 (0.931, 1.209)
35–37	0.324	0.345 (0.072)	1.064 (0.930, 1.199)	0.257 (0.069)	0.792 (0.692, 0.893)
38–39	0.328	0.236 (0.055)	0.721 (0.627, 0.815)	0.187 (0.052)	0.569 (0.494, 0.645)
40–42	0.328	0.300 (0.068)	0.914 (0.826, 1.002)	0.123 (0.045)	0.376 (0.339, 0.412)
43–44	0.344	0.253 (0.080)	0.736 (0.656, 0.815)	0.068 (0.027)	0.197 (0.175, 0.219)
45–50	0.327	0.276 (0.117)	0.844 (0.766, 0.921)	0.026 (0.017)	0.079 (0.071, 0.086)
		***p* < 0.001**		***p* < 0.001**	
Source of egg					
Patient	0.254	0.255 (0.099)	1.005 (0.996, 1.014)	0.260 (0.094)	1.023 (1.013, 1.032)
Donor	0.326	0.287 (0.090)	0.879 (0.839, 0.919)	0.141 (0.112)	0.433 (0.411, 0.455)
		***p* < 0.001**		***p* < 0.001**	
Duration of infertility (years)					
0	0.267	0.330 (0.118)	1.237 (1.167, 1.308)	0.264 (0.105)	0.988 (0.932, 1.044)
1–3	0.269	0.271 (0.103)	1.007 (0.994, 1.020)	0.281 (0.093)	1.044 (1.030, 1.057)
4–6	0.259	0.249 (0.089)	0.962 (0.947, 0.977)	0.237 (0.088)	0.918 (0.904, 0.932)
7–9	0.229	0.236 (0.093)	1.032 (1.001, 1.062)	0.242 (0.104)	1.060 (1.028, 1.091)
10–12	0.212	0.211 (0.086)	0.998 (0.949, 1.048)	0.230 (0.104)	1.088 (1.033, 1.143)
13-	0.181	0.191 (0.090)	1.055 (0.981, 1.130)	0.164 (0.092)	0.910 (0.845, 0.975)
		***p* < 0.001**		***p* < 0.001**	
Previous unsuccessful IVF cycles					
0	0.275	0.289 (0.094)	1.050 (1.039, 1.061)	0.280 (0.096)	1.017 (1.006, 1.028)
1	0.224	0.205 (0.079)	0.915 (0.895, 0.936)	0.232 (0.083)	1.037 (1.014, 1.061)
2	0.227	0.196 (0.075)	0.861 (0.833, 0.889)	0.209 (0.080)	0.920 (0.890, 0.951)
3+	0.211	0.181 (0.080)	0.859 (0.827, 0.891)	0.173 (0.077)	0.821 (0.791, 0.852)
		***p* < 0.001**		***p* < 0.001**	
Previous live birth by IVF					
No	0.253	0.251 (0.096)	0.993 (0.983, 1.003)	0.246 (0.086)	0.975 (0.965, 0.985)
Yes	0.272	0.280 (0.111)	1.028 (1.007, 1.049)	0.301 (0.127)	1.105 (1.082, 1.128)
		*p* = 0.01		***p* < 0.001**	
Treatment type					
IVF	0.234	0.234 (0.091)	1.001 (0.987, 1.015)	0.249 (0.102)	1.065 (1.050, 1.080)
ICSI	0.277	0.277 (0.095)	0.999 (0.987, 1.011)	0.263 (0.098)	0.949 (0.938, 0.960)
		*p* = 0.77		***p* < 0.001**	
Cause of infertility					
Unexplained	0.242	0.244 (0.102)	1.007 (0.991, 1.023)	0.237 (0.098)	0.980 (0.964, 0.995)
Tubal only	0.237	0.233 (0.085)	0.982 (0.955, 1.010)	0.255 (0.093)	1.074 (1.044, 1.104)
Ovulatory only	0.281	0.267 (0.093)	0.950 (0.918, 0.982)	0.266 (0.098)	0.949 (0.917, 0.981)
Endometriosis only	0.253	0.253 (0.085)	0.998 (0.952, 1.043)	0.261 (0.086)	1.029 (0.982, 1.077)
Male cause only	0.271	0.274 (0.102)	1.011 (0.995, 1.026)	0.271 (0.097)	1.002 (0.987, 1.017)
Multiple causes	0.264	0.264 (0.094)	0.997 (0.968, 1.026)	0.266 (0.092)	1.006 (0.977, 1.035)
		*p* = 0.09		***p* < 0.001**	

P-values are for differences between observed and predicted number of live births, and those smaller than the threshold for a family-wise error rate of 5% are highlighted.

* Not including 5 cycles where cervical cause of infertility only, for which meaningful confidence intervals cannot be calculated.

### Overall performance

Brier scores for all four models (IVFpredict and Templeton, original and updated) are shown in Table 4. The original and updated IVFpredict models had smaller Brier scores than the Templeton model, indicating better predictive accuracy. The updated models had better reliability than the original models, with IVFpredict having the best reliability. The IVFpredict models also had higher resolution.

**Table 4:** Brier scores and decomposition for IVFpredict and Templeton models, from 130,960 IVF cycles.

	Scaled modified (Sanders) Brier score	Scaled reliability (in-the-small)	Scaled uncertainty
Templeton model			
Original	1.065	0.096	0.032
Updated	0.971	0.003	0.032
IVFpredict			
Original	0.965	0.004	0.039
Updated	0.963	0.001	0.039

Scaled modified Brier score = 1 + Scaled reliability—Scaled uncertainty.

The rates in Table 5 show that, before updating, the Templeton model gives a lower probability of live birth than IVFpredict in 128,615 cycles (98.2%), as the Templeton model severely underestimates the probability of live birth. Thus the Templeton model gave a more accurate prognosis in cycles that did not result in a live birth and IVFpredict gave a more accurate prognosis in the fewer cycles that resulted in a live birth. This comparison is much more meaningful after updating both models. After updating, most women who had a live birth were given a higher probability of live birth by IVFpredict than the Templeton model, and most women who did not have a live birth were given a lower probability of live birth by IVFpredict. I.e., after updating, IVFpredict performed better than the Templeton model in terms of correctly predicting both having a live birth and not having a live birth. The net reclassification index was positive, favouring IVFpredict over the Templeton model, for both the original and updated models, being considerably higher for the updated models.

**Table 5:** Net reclassification index for IVFpredict compared with the Templeton model.

Original				
Prognosis	No live birth		Live birth	
IVFpredict higher than Templeton	95,304	(97.8%)	33,311	(99.3%)
Templeton higher than IVFpredict	2,103	(2.2%)	242	(0.7%)
Net reclassification index	2.88% from Templeton to IVFpredict			
Updated*				
Prognosis	No live birth		Live birth	
IVFpredict higher than Templeton	46,750	(48.0%)	18,143	(54.1%)
Templeton higher than IVFpredict	50,654	(52.0%)	15,410	(45.9%)
Net reclassification index	12.2% from Templeton to IVFpredict			

* Not including 3 cycles with equal prognosis in the updated IVFpredict and Templeton models.

## Discussion

In this study we have shown that IVFpredict externally validates and has good discrimination and calibration in a large independent cohort. We have further shown that it has better discrimination and calibration than the Templeton model for accurately predicting live birth. This is the first time that IVFpredict has been validated for live birth outcome on a different sample from that used for its development. The observed lower discrimination in comparison to the original development sample is to be expected [21,29], but the magnitude of the difference is tiny (mean difference of 0.01) and the observed AUROC of 0.628 in this validation sample can be thought of as excellent discriminative performance, as it is higher than the supposed maximum of 0.62 expected for a prediction model of IVF success [23]. Female age is the most important predictor of live birth from IVF [30], and IVFpredict has the drawback that it considers age in categories rather than a continuous measure. However, we demonstrated that IVFpredict has better discriminatory power than any model based only on a monotone transformation of female age, showing that the additional predictors used by IVFpredict give additional discriminatory power. As this was a validation study, it was not appropriate to investigate how prediction models could be improved by the inclusion of additional variables.

The overall better performance, in terms of discrimination and calibration, of IVFpredict in comparison with the Templeton model in this study, as in our original development cohort, is in contrast to the conclusions of a recent Dutch study that reported similar discrimination and calibration of the two models [18]. However, the differences between AUROC and calibration for the two models in that study were minimal and, as discussed in the introduction, the Dutch study was small, included only treatment cycles in couples with primary infertility, could not include information on ICSI or whether women used their own or donor eggs and had pregnancy (rather than live birth) as the measured outcome. Our finding, that the Templeton model underestimated the live birth rate by up to 17% in the current study, is consistent with other studies that have previously demonstrated that the Templeton model was particularly poorly calibrated when applied to contemporary cohorts [15,17]. The likely explanation for this is the increase in the use and success of IVF in the two decades since the Templeton model was originally developed. However, IVFpredict had better calibration even after both models were updated to take account of the live birth rate in the contemporary UK cohort used here. This is likely to be because the Templeton model does not consider ICSI and donor eggs as informative predictors of prognosis and therefore incorrectly estimates, sometimes extremely, the probability of live birth in cycles using these treatment methods.

Our preferred method of updating the prediction models to account for the differences in IVF success in different populations caused the predicted live birth rate to equal the observed live birth rate in the whole validation sample, therefore removing the systematic underestimation exhibited by both models. The updated IVFpredict model had a calibration plot slope closer to the target value than that of the Templeton model, and a better reliability in the Brier score decomposition. Furthermore, the updated Templeton model overestimated the probability of live birth in older women using their own eggs and women with a tubal cause of infertility, and underestimated the probability of live birth from cycles using ICSI and women who had not had a previous live birth from IVF. Both updated models underestimated the probability of live birth in cycles using donor eggs, but this was more marked in the updated Templeton model. The updated IVFpredict model had a smaller Brier score, indicating a better overall calibration, and in particular showed greater resolution in the Brier score decomposition. This shows that IVFpredict has the desirable property of covering a greater range of probabilities than the Templeton model [23], indicating that it carries more predictive prognostic information [31]. Consistent with this, the updated IVFpredict model provided a more accurate prognosis than the updated Templeton model for the majority (52.5%) of the cycles in the validation sample.

Our results suggest that, whichever model is chosen for use in clinical practice, it should be updated or recalibrated before it is used to inform patients of their prognosis. This validation study has shown the potential for extremely misleading predictions if a poorly calibrated, out of date model is used. Ideally recalibration should take place on a per-centre basis due to the amount of variation in the live birth rate between treatment centres [15,30,32]. The simplest method of recalibration would involve a treatment centre retrospectively applying a prediction model to their recorded cycles, and calculating how much to add or subtract from the linear predictor to ensure that the average predicted live birth rate equals the observed live birth rate at that centre. Larger centres could create calibration plots, as in Fig. 2, and use this as described above to convert a probability given by the prediction model for certain patient characteristics into a centre-specific probability for that patient. Unfortunately the HFEA dataset used here did not contain centre details for each cycle, so we could not assess the efficacy of per-centre recalibration.

This study benefits from a large validation sample that is highly representative of the target population. It should be noted that, since both the IVFpredict and Templeton models were developed using UK HFEA data, we were only able to perform temporal validation [30]. External validation of the ability of IVFpredict to predict live birth rate in couples with both primary and secondary infertility in a large non-UK population is required to assess its global applicability. Neither IVFpredict nor the Templeton model consider live birth from frozen embryo replacements, hence both models may be disadvantaged given the likelihood of increased use of frozen embryo replacements. No prediction models for live birth after IVF have yet reached the impact analysis stage and external validation is an essential step towards this [1]. In conclusion, this validation study indicates that IVFpredict has superior discrimination and calibration to the Templeton model for informing patients and clinicians in the UK of the prognosis of live birth following IVF. IVFpredict exhibits improved calibration after updating, and recalibration can be applied on a per-centre basis in clinical practice.

## Supporting Information

S1 Table Figures for calibration plot for the IVFpredict and Templeton models. Based on 130,960 IVF cycles.
(DOCX)

S2 Table Figures for calibration plot for updated IVFpredict and Templeton models. Based on 130,960 IVF cycles.
(DOCX)

S3 Table Observed and predicted live birth rates from the IVFpredict and Templeton models. Stratified by characteristics of patients and treatment, in 130,960 IVF cycles.
(DOCX)

## References

[1] Leushuis E, van der Steeg JW, Steures P, Bossuyt PMM, Eijkemans MJC, van der Veen, et al Prediction models in reproductive medicine: a critical appraisal. Hum Reprod Update 2009;15: 537–552. 10.1093/humupd/dmp013 19435779

[2] Hemingway H, Croft P, Perel P, Hayden JA, Abrams K, Timmis A, et al Prognosis research strategy (PROGRESS) 1: a framework for researching clinical outcomes. BMJ 2013;346: e5595 10.1136/bmj.e5595 23386360 PMC3565687

[3] Steyerberg EW, Moons KGM, van der Windt DA, Hayden JA, Perel P, Schroter S, et al Prognosis research strategy (PROGRESS) 3: prognostic model research. PLOS Med 2013;10: e1001381 10.1371/journal.pmed.1001381 23393430 PMC3564751

[4] Weigerinck MAHM, Bongers MY, Mol BWJ, Heineman M. How concordant are the estimated rates of natural conception and in-vitro fertilization/embryo transfer success? Hum Reprod 1999;14: 689–693. 10221696 10.1093/humrep/14.3.689

[5] van der Steeg JW, Steures P, Eijkemans MJC, Habbema JDF, Bossuyt PMM, Hompes PGA, et al Do clinical prediction models improve concordance of treatment decisions in reproductive medicine? BJOG 2006;113: 825–831. 16827767 10.1111/j.1471-0528.2006.00992.x

[6] Bouckaert A, Psalti I, Loumaye E, De Cooman S, Thomas K. The probability of a successful treatment of infertility by in-vitro fertilization. Hum Reprod 1994;9: 448–455. 8006133 10.1093/oxfordjournals.humrep.a138526

[7] Templeton A, Morris JK, Parslow W. Factors that affect outcome of in-vitro fertilisation treatment. Lancet 1996;348: 1402–1406. 8937279 10.1016/S0140-6736(96)05291-9

[8] Minaretzis D, Harris D, Alper MM, Mortola JF, Berger MJ, Power D. Multivariate analysis of factors predictive of successful live births in in vitro fertilization (IVF) suggests strategies to improve IVF outcome. J Assist Reprod Genet 1998;15: 365–371. 9673880 10.1023/A:1022528915761 PMC3455016

[9] Strandell A, Bergh C, Lundin K. Selection of patients suitable for one-embryo transfer may reduce the rate of multiple births by half without impairment of overall birth rates. Hum Reprod 2000;15: 2520–2525. 11098020 10.1093/humrep/15.12.2520

[10] Wang YA, Healy D, Black D, Sullivan EA. Age-specific success rate for women undertaking their first assisted reproductive technology treatment using their own oocytes in Australia, 2002–2005. Hum Reprod 2008;23: 1633–1638. 10.1093/humrep/den135 18441345

[11] Banerjee P, Choi B, Shahine LK, Jun SH, O’Leary K, Lathri RB, et al Deep phenotying to predict live birth outcomes in in vitro fertilization. Proc Natl Acad Sci U S A 2010;107: 13570–13575. 10.1073/pnas.1002296107 20643955 PMC2922227

[12] Nelson SM, Lawlor DA. Predicting live birth, preterm delivery, and low birth weight in infants born from in vitro fertilisation: a prospective study of 144,018 treatment cycles. PLOS Med 2011;8: e1000386 10.1371/journal.pmed.1000386 21245905 PMC3014925

[13] Jones CA, Christensen AL, Salihu H, Carpenter W, Petrozzino J, Abrams E, et al Prediction of individual probabilities of livebirth and multiple birth events following in vitro fertilization (IVF): a new outcomes counselling tool for IVF providers and patients using HFEA metrics. J Exp Clin Assist Reprod 2011;8: 3 21991292 PMC3183499

[14] Li HWR, Lee VCY, Lau EYL, Yeung WSB, Ho PC, Ng EHY. Role of baseline antral follicle count and anti-Mullerian hormone in prediction of cumulative live birth in the first in vitro fertilisation cycle: a retrospective cohort analysis. PLOS ONE 2013;8: e61095 10.1371/journal.pone.0061095 23637787 PMC3634063

[15] Arvis P, Lehert P, Guivarc’h-Levêque A. Simple adaptations to the Templeton model for IVF outcome prediction make it current and clinically useful. Hum Reprod 2012;10: 2971–2978. 10.1093/humrep/des283 22851717

[16] Smeenk JMJ, Stolwijk AM, Kremer JAM, Braat DDM. External validation of the Templeton model for predicting success after IVF. Hum Reprod 2000;15: 1065–1068. 10783353 10.1093/humrep/15.5.1065

[17] van Loendersloot LL, van Wely M, Repping S, van der Veen F, Bossuyt PMM. Templeton prediction model underestimates IVF success in an external validation. Reprod Biomed Online 2011;22: 597–602. 10.1016/j.rbmo.2011.02.012 21493154

[18] te Velde ER, Nieboer D, Lintsen AM, Braat DDM, Eijkemans MJC, Habbema JDF, et al Comparison of two models predicting IVF success; the effect of time trends on model performance. Hum Reprod 2014;29: 57–64. 10.1093/humrep/det393 24242632

[19] Palermo G, Joris H, Devroey P, van Steirteghem AC. Pregnancies after intracytoplasmic injection of single spermatozoon into an oocyte. Lancet 1992;340: 17–18. 1351601 10.1016/0140-6736(92)92425-f

[20] Ferraretti AP, Goossens V, de Mouzon J, Bhattacharya S, Castilla JA, Korsak V, et al Assisted reproductive technology in Europe, 2008: results generated from European registers by ESHRE. Hum Reprod 2012;27: 2571–2584. 22786779 10.1093/humrep/des255

[21] Bouwmeester W, Zuithoff NPA, Mallett S, Geerlings MI, Vergouwe Y, Steyerberg EW, et al Reporting and methods in clinical prediction research: a systematic review. PLOS Med 2012;9: e1001221. 10.1371/journal.pmed.1001221 PMC3358324 22629234

[22] Cook NR. Statistical evaluation of prognostic versus diagnostic models: beyond the ROC curve. Clin Chem 2008;54: 17–23. 18024533 10.1373/clinchem.2007.096529

[23] Coppus SFPJ, van der Veen F, Opmeer BC, Mol BWJ, Bossuyt PMM. Evaluating prediction models in reproductive medicine. Hum Reprod 2009;24: 1774–1778. 10.1093/humrep/dep109 19395365

[24] Macklon NS, Stouffer RL, Guidice LC, Fauser BCJM. The science behind 25 years of ovarian stimulation for in vitro fertilization. Endocr Rev 2006;27: 170–207. 16434510 10.1210/er.2005-0015

[25] Hosmer DW, Lemeshow S. Applied Logistic Regression. 2nd ed. New York: John Wiley & Sons; 2000.

[26] Wilks DS. Statistical Methods in the Atmospheric Sciences. Burlington, MA: Elsevier; 2006.

[27] Pencina MJ, D’Agostino RB Sr, D’Agostino RB Jr, Vasan RS. Evaluating the added predictive ability of a new marker: from area under the ROC curve to reclassification and beyond. Stat Med 2008;27: 157–172. 17569110 10.1002/sim.2929

[28] Janssen KJM, Vergouwe Y, Kalkman CJ, Grobbee DE, Moons KGM. A simple method to adjust clinical prediction models to local circumstances. Can J Anaesth 2009;56: 194–201. 10.1007/s12630-009-9041-x 19247740 PMC5487883

[29] Moons KGM, Kengne AP, Grobbee DE, Royston P, Vergouwe Y, Altman DG, et al Risk prediction models: II. External validation, model updating, and impact assessment. Heart 2012;98: 691–698. 10.1136/heartjnl-2011-301247 22397946

[30] van Loendersloot LL, Repping S, Bossuyt PMM, van der Veen F, van Wely M. Prediction models in in vitro fertilization; where are we? A mini review. J Advanc Res 2014;5: 295–301. 10.1016/j.jare.2013.05.002 PMC4294714 25685496

[31] Altman DG, Royston P. What do we mean by validating a prognostic model? Stat Med 2000;19: 453–473. 10694730 10.1002/(sici)1097-0258(20000229)19:4<453::aid-sim350>3.0.co;2-5

[32] Lintsen AME, Braat DDM, Habbema JDF, Kremer JAM, Eijkemans MJC. Can differences in IVF success rates between centres be explained by patient characteristics and sample size? Hum Reprod 2010;25: 110–117. 10.1093/humrep/dep358 19837684

