# Supplementary material for: External Validation and Calibration of IVFpredict: A National Prospective Cohort Study of 130,960 In Vitro Fertilisation Cycles
Source: PLoS One. 2015 Apr 8;10(4):e0121357. doi: 10.1371/journal.pone.0121357 (PMC4390202; doi:10.1371/journal.pone.0121357)
Supplement: S1 Table — Based on 130,960 IVF cycles. (DOCX) [file pone.0121357.s001.docx]

**S1 Table. Figures for calibration plot for the IVFpredict and Templeton models.** Based on 130,960 IVF cycles.

|  | IVFpredict | | | Templeton | | |
| --- | --- | --- | --- | --- | --- | --- |
| Decile | Observed live birth rate | Predicted live birth rate (SD) | Difference | Observed live birth rate | Predicted live birth rate (SD) | Difference |
| 1 | 0.087 | 0.064 (0.026) | -0.023 | 0.115 | 0.034 (0.013) | -0.081 |
| 2 | 0.147 | 0.125 (0.012) | -0.022 | 0.152 | 0.065 (0.007) | -0.087 |
| 3 | 0.206 | 0.165 (0.011) | -0.041 | 0.197 | 0.086 (0.005) | -0.111 |
| 4 | 0.230 | 0.199 (0.009) | -0.031 | 0.236 | 0.103 (0.005) | -0.133 |
| 5 | 0.262 | 0.225 (0.008) | -0.037 | 0.266 | 0.118 (0.004) | -0.148 |
| 6 | 0.294 | 0.254 (0.008) | -0.040 | 0.283 | 0.132 (0.003) | -0.151 |
| 7 | 0.294 | 0.277 (0.006) | -0.017 | 0.300 | 0.144 (0.004) | -0.156 |
| 8 | 0.323 | 0.302 (0.007) | -0.021 | 0.315 | 0.161 (0.005) | -0.154 |
| 9 | 0.343 | 0.333 (0.010) | -0.010 | 0.344 | 0.177 (0.004) | -0.167 |
| 10 | 0.377 | 0.374 (0.025) | -0.003 | 0.360 | 0.223 (0.042) | -0.137 |
| Hosmer-Lemeshow test |  | *p* < 0.001 |  |  | *p* < 0.001 |  |
| Intercept (95% CI) |  | 0.040 (0.017, 0.063) |  |  | 0.080 (0.044, 0.117) |  |
| Slope (95% CI) |  | 0.932 (0.839, 1.025) |  |  | 1.419 (1.149, 1.690) |  |
