# Supplementary material for: External Validation and Calibration of IVFpredict: A National Prospective Cohort Study of 130,960 In Vitro Fertilisation Cycles
Source: PLoS One. 2015 Apr 8;10(4):e0121357. doi: 10.1371/journal.pone.0121357 (PMC4390202; doi:10.1371/journal.pone.0121357)
Supplement: S2 Table — Based on 130,960 IVF cycles. (DOCX) [file pone.0121357.s002.docx]

**S2 Table. Figures for calibration plot for the updated IVFpredict and Templeton models.** Based on 130,960 IVF cycles.

|  | IVFpredict | | | Templeton | | |
| --- | --- | --- | --- | --- | --- | --- |
| Decile | Observed live birth rate | Predicted live birth rate (SD) | Difference | Observed live birth rate | Predicted live birth rate (SD) | Difference |
| 1 | 0.087 | 0.073 (0.030) | -0.014 | 0.115 | 0.081 (0.030) | -0.034 |
| 2 | 0.147 | 0.142 (0.013) | -0.003 | 0.152 | 0.150 (0.014) | -0.002 |
| 3 | 0.206 | 0.186 (0.012) | -0.020 | 0.197 | 0.191 (0.011) | -0.006 |
| 4 | 0.230 | 0.222 (0.009) | -0.008 | 0.236 | 0.224 (0.009) | -0.012 |
| 5 | 0.262 | 0.251 (0.009) | -0.011 | 0.266 | 0.253 (0.007) | -0.013 |
| 6 | 0.294 | 0.281 (0.008) | -0.013 | 0.283 | 0.277 (0.006) | -0.006 |
| 7 | 0.294 | 0.306 (0.007) | +0.012 | 0.300 | 0.298 (0.007) | -0.002 |
| 8 | 0.323 | 0.332 (0.008) | +0.009 | 0.315 | 0.326 (0.009) | +0.011 |
| 9 | 0.343 | 0.365 (0.010) | +0.022 | 0.344 | 0.352 (0.007) | +0.008 |
| 10 | 0.377 | 0.407 (0.026) | +0.030 | 0.360 | 0.417 (0.056) | +0.057 |
| Hosmer-Lemeshow test |  | *p* < 0.001 |  |  | *p* < 0.001 |  |
| Intercept (95% CI) |  | 0.034 (0.012, 0.055) |  |  | 0.050 (0.021, 0.080) |  |
| Slope (95% CI) |  | 0.867 (0.789, 0.946) |  |  | 0.804 (0.697,0.912) |  |
