# Supplementary material for: External Validation and Calibration of IVFpredict: A National Prospective Cohort Study of 130,960 In Vitro Fertilisation Cycles
Source: PLoS One. 2015 Apr 8;10(4):e0121357. doi: 10.1371/journal.pone.0121357 (PMC4390202; doi:10.1371/journal.pone.0121357)
Supplement: S3 Table — Stratified by characteristics of patients and treatment, in 130,960 IVF cycles. (DOCX) [file pone.0121357.s003.docx]

**S3 Table. Observed and predicted live birth rates from the IVFpredict and Templeton models.** Stratified by characteristics of patients and treatment, in 130,960 IVF cycles.

|  |  | IVFpredict | | Templeton | |
| --- | --- | --- | --- | --- | --- |
|  | Observed live birth rate | Predicted live birth rate (SD) | Ratio predicted to observed (95% CI) | Predicted live birth rate (SD) | Ratio predicted to observed (95% CI) |
| All patients | 0.256 | 0.232 (0.092) | 0.905 (0.896, 0.913) | 0.124 (0.055) | 0.483 (0.479, 0.487) |
| Female age (years) patient eggs |  |  |  |  |  |
| 18-34 | 0.326 | 0.300 (0.057) | 0.922 (0.910, 0.933) | 0.163 (0.039) | 0.500 (0.494, 0.506) |
| 35-37 | 0.273 | 0.246 (0.053) | 0.902 (0.886, 0.918) | 0.131 (0.040) | 0.479 (0.470, 0.488) |
| 38-39 | 0.198 | 0.180 (0.043) | 0.912 (0.887, 0.937) | 0.097 (0.032) | 0.488 (0.475, 0.502) |
| 40-42 | 0.129 | 0.106 (0.030) | 0.840 (0.808, 0.873) | 0.065 (0.026) | 0.502 (0.483, 0.521) |
| 43-44 | 0.050 | 0.040 (0.014) | 0.794 (0.688, 0.901) | 0.033 (0.013) | 0.659 (0.570, 0.747) |
| 45-50 | 0.020 | 0.019 (0.012) | 0.978 (0.551, 1.405) | 0.015 (0.007) | 0.742 (0.419, 1.066) |
| Female age (years) donor eggs |  |  |  |  |  |
| 18-34 | 0.301 | 0.288 (0.063) | 0.958 (0.834, 1.082) | 0.160 (0.042) | 0.533 (0.463, 0.602) |
| 35-37 | 0.324 | 0.315 (0.069) | 0.971 (0.849, 1.094) | 0.122 (0.041) | 0.377 (0.329, 0.425) |
| 38-39 | 0.328 | 0.212 (0.051) | 0.648 (0.563, 0.733) | 0.084 (0.028) | 0.257 (0.223, 0.291) |
| 40-42 | 0.328 | 0.272 (0.065) | 0.829 (0.749, 0.909) | 0.053 (0.022) | 0.163 (0.146, 0.179) |
| 43-44 | 0.344 | 0.228 (0.075) | 0.664 (0.592, 0.736) | 0.028 (0.012) | 0.082 (0.073, 0.091) |
| 45-50 | 0.327 | 0.250 (0.111) | 0.766 (0.695, 0.837) | 0.010 (0.007) | 0.032 (0.029, 0.035) |
| Source of egg |  |  |  |  |  |
| Patient | 0.254 | 0.231 (0.092) | 0.909 (0.900, 0.917) | 0.126 (0.054) | 0.494 (0.490, 0.499) |
| Donor | 0.326 | 0.260 (0.085) | 0.797 (0.761, 0.833) | 0.065 (0.058) | 0.201 (0.190, 0.211) |
| Duration of infertility (years) |  |  |  |  |  |
| 0 | 0.267 | 0.302 (0.111) | 1.131 (1.067, 1.195) | 0.129 (0.059) | 0.482 (0.454, 0.509) |
| 1-3 | 0.269 | 0.245 (0.096) | 0.912 (0.901, 0.924) | 0.137 (0.053) | 0.511 (0.504, 0.517) |
| 4-6 | 0.259 | 0.224 (0.082) | 0.868 (0.855, 0.882) | 0.113 (0.049) | 0.436 (0.429, 0.443) |
| 7-9 | 0.229 | 0.213 (0.086) | 0.930 (0.903, 0.958) | 0.117 (0.060) | 0.511 (0.495, 0.526) |
| 10-12 | 0.212 | 0.190 (0.079) | 0.897 (0.852, 0.942) | 0.110 (0.059) | 0.512 (0.494, 0.547) |
| 13- | 0.181 | 0.171 (0.082) | 0.947 (0.880, 1.013) | 0.075 (0.048) | 0.416 (0.386, 0.446) |
| Previous unsuccessful IVF cycles |  |  |  |  |  |
| 0 | 0.275 | 0.262 (0.088) | 0.953 (0.943, 0.963) | 0.137 (0.056) | 0.498 (0.493, 0.504) |
| 1 | 0.224 | 0.184 (0.072) | 0.821 (0.803, 0.840) | 0.110 (0.044) | 0.489 (0.478, 0.500) |
| 2 | 0.227 | 0.175 (0.069) | 0.772 (0.747, 0.797) | 0.097 (0.041) | 0.427 (0.413, 0.441) |
| 3+ | 0.211 | 0.162 (0.073) | 0.769 (0.740, 0.797) | 0.078 (0.038) | 0.372 (0.359, 0.386) |
| Previous live birth by IVF |  |  |  |  |  |
| No | 0.253 | 0.227 (0.089) | 0.898 (0.889, 0.907) | 0.117 (0.046) | 0.465 (0.460, 0.469) |
| Yes | 0.272 | 0.254 (0.103) | 0.934 (0.915, 0.953) | 0.153 (0.077) | 0.561 (0.549, 0.572) |
| Treatment type |  |  |  |  |  |
| IVF | 0.234 | 0.211 (0.084) | 0.902 (0.890, 0.915) | 0.119 (0.053) | 0.511 (0.504, 0.518) |
| ICSI | 0.277 | 0.251 (0.095) | 0.906 (0.895, 0.917) | 0.128 (0.056) | 0.461 (0.455, 0.466) |
| Cause of infertility |  |  |  |  |  |
| Unexplained | 0.242 | 0.220 (0.094) | 0.909 (0.895, 0.924) | 0.113 (0.053) | 0.468 (0.460, 0.475) |
| Tubal only | 0.237 | 0.210 (0.079) | 0.885 (0.860, 0.909) | 0.123 (0.053) | 0.517 (0.503, 0.532) |
| Ovulatory only | 0.281 | 0.241 (0.086) | 0.860 (0.831, 0.889) | 0.129 (0.055) | 0.461 (0.446, 0.477) |
| Endometriosis only | 0.253 | 0.228 (0.078) | 0.901 (0.589, 0.942) | 0.125 (0.049) | 0.495 (0.472, 0.518) |
| Male cause only | 0.271 | 0.248 (0.095) | 0.916 (0.902, 0.930) | 0.132 (0.056) | 0.489 (0.481, 0.496) |
| Multiple causes | 0.264 | 0.239 (0.087) | 0.902 (0.876, 0.928) | 0.129 (0.052) | 0.487 (0.473, 0.502) |

* Not including 5 cycles where cervical cause of infertility only, for which meaningful confidence intervals cannot be calculated.
